# Spatial Statistics and Influencing Factors of the COVID-19 Epidemic at Both Prefecture and County Levels in Hubei Province, China

**DOI:** 10.3390/ijerph17113903

**Published:** 2020-05-31

**Authors:** Yongzhu Xiong, Yunpeng Wang, Feng Chen, Mingyong Zhu

**Affiliations:** 1 School of Geography and Tourism, Jiaying University, Meizhou 514015, China; 2 Guangzhou Institute of Geochemistry, Chinese Academy of Sciences, Guangzhou 510640, China; wangyp@gig.ac.cn; 3 College of Computer and Information Engineering, Xiamen University of Technology, Xiamen 361024, China; chenfeng@xmut.edu.cn; 4 Big Data Institute of Digital Natural Disaster Monitoring in Fujian, Xiamen University of Technology, Xiamen 361024, China

**Keywords:** COVID-19, spatial scale, influencing factor, spatial autocorrelation, Spearman’s rank correlation, Wuhan city

## Abstract

The coronavirus disease 2019 (COVID-19) epidemic has had a crucial influence on people’s lives and socio-economic development. An understanding of the spatiotemporal patterns and influencing factors of the COVID-19 epidemic on multiple scales could benefit the control of the outbreak. Therefore, we used spatial autocorrelation and Spearman’s rank correlation methods to investigate these two topics, respectively. The COVID-19 epidemic data reported publicly and relevant open data in Hubei province were analyzed. The results showed that (1) at both prefecture and county levels, the global spatial autocorrelation was extremely significant for the cumulative confirmed COVID-19 cases (CCC) in Hubei province from 30 January to 18 February 2020. Further, (2) at both levels, the significant hotspots and cluster/outlier areas were observed solely in Wuhan city and most of its districts/sub-cities from 30 January to 18 February 2020. (3) At the prefecture level in Hubei province, the number of CCC had a positive and extremely significant correlation (*p* < 0.01) with the registered population (RGP), resident population (RSP), Baidu migration index (BMI), regional gross domestic production (GDP), and total retail sales of consumer goods (TRS), respectively, from 29 January to 18 February 2020 and had a negative and significant correlation (*p* < 0.05) with minimum elevation (MINE) from 2 February to 18 February 2020, but no association with the land area (LA), population density (PD), maximum elevation (MAXE), mean elevation (MNE), and range of elevation (RAE) from 23 January to 18 February 2020. (4) At the county level, the number of CCC in Hubei province had a positive and extremely significant correlation (*p* < 0.01) with PD, RGP, RSP, GDP, and TRS, respectively, from 27 January to 18 February 2020, and was negatively associated with MINE, MAXE, MNE, and RAE, respectively, from 26 January to 18 February 2020, and negatively associated with LA from 30 January to 18 February 2020. It suggested that (1) the COVID-19 epidemics at both levels in Hubei province had evident characteristics of significant global spatial autocorrelations and significant centralized high-risk outbreaks. (2) The COVID-19 epidemics were significantly associated with the natural factors, such as LA, MAXE, MNE, and RAE, -only at the county level, not at the prefecture level, from 2 February to 18 February 2020. (3) The COVID-19 epidemics were significantly related to the socioeconomic factors, such as RGP, RSP, TRS, and GDP, at both levels from 26 January to 18 February 2020. It is desired that this study enrich our understanding of the spatiotemporal patterns and influencing factors of the COVID-19 epidemic and benefit classified prevention and control of the COVID-19 epidemic for policymakers.

## 1. Introduction

Since December 2019, coronavirus disease 2019 (COVID-19) emerged in Wuhan city, then rapidly spread throughout China [1]. Chinese health authorities did an immediate investigation to characterise and control the disease [2]. During the early outbreak of the COVID-19 epidemic in China, large population movements and gathering could have intensified the transmission of the novel coronavirus SARS-CoV-2 and the spread of the epidemic, posing a severe threat to human lives and public health. Chinese governments at all levels have made rapid response schemes to the COVID-19 epidemic and have taken urgent steps and effective measures to prevent the epidemic spread within China. This is the first time Chinese authorities have shut down transportation and travel in and out of Wuhan, which has been proven effective in preventing and controlling the spread of the COVID-19 epidemic in practice.

A cumulative total of 82,802 confirmed COVID-19 cases, including 3331 deaths, was reported in China as of 2 April 2020 [3]. On the same day, more than one million of cumulative confirmed COVID-19 cases (CCC), including 52,973 deaths worldwide, were tracked and mapped online at the Johns Hopkins COVID-19 map dashboard [4]. As of 15 April 2020, the number of CCC worldwide rapidly rose to more than two million, causing more than 127,000 deaths. At present, the situation of the COVID-19 epidemic in China has been under control and has dramatically improved. However, the global COVID-19 pandemics have been drastically deteriorating due to the rapid growth of confirmed COVID-19 cases worldwide. It is essential to support global cooperation and collaboration for the prevention and control of the pandemic [5], and it is also crucial to make reasonable policies from the country to the county facing the COVID-19 pandemic.

Shortly after the COVID-19 outbreak, some scholars carried out extensive research on COVID-19 in terms of pathogenesis, epidemiology, etiology, biology, virology, molecular biology, genomics, imaging, and clinical medicine [1,6,7,8,9,10,11,12,13,14,15,16,17,18,19,20] as well as spread modeling [21,22,23]. Their research results have provided critical scientific bases for the diagnosis and treatment of COVID-19 and the prevention and control of the COVID-19 epidemic. Furthermore, some universities, research institutions, and online platforms have launched daily updates of the dynamic epidemic information based on epidemic maps and statistical data [4], which have provided essential information support for governments and the public to understand the progress of the epidemic quickly and intuitively. However, these updates cannot offer an insight into the spatiotemporal patterns and influencing factors of the COVID-19 epidemic, which is very important for the prevention and control of the epidemic.

Time-series spatial statistics and influencing factor analyses can help unveil the spatiotemporal characteristics and evolution mechanism of the epidemic, consequently assisting scientific decision making for the prevention and control of the epidemic. Previous studies investigated the transmission network [24], spatial correlation [25,26,27], space–time transmission dynamics [28], and spatiotemporal characteristics of the 2003 SARS (severe acute respiratory syndrome) epidemic [29], providing a useful reference for the study of spatial statistics of the COVID-19 epidemic. Previous studies showed that the characteristics of gathering and spreading of major infectious diseases like SARS 2003 had significantly positive autocorrelations and spatial clusters. COVID-19 is a SARS-like infectious disease that mainly achieves human-to-human transmission through respiratory droplets, person-to-person contact, and fecal-mouth contact, with a median incubation period of four days [1]. Recently, Kang et al. (2020) [30] described the spatiotemporal pattern and measured the spatial association of the provincial COVID-19 epidemic in mainland China from 16 January to 6 February 2020. Their results showed that the COVID-19 infections had a significant spatial association, in agreement with the 2003 SARS results of Hu et al. (2013) [31]. Inspired by previous studies, we hypothesized that spatiotemporal patterns of the COVID-19 epidemic should have significant autocorrelations and spatial clusters, similar to SARS 2003, on multiple scales.

As for the influencing factors of the COVID-19 epidemic, previous literature showed that meteorological variables such as temperature and humidity on the large spatial scales were mainly investigated. Sun et al. (2020) [32] proposed that natural (cold temperature and low humidity) and social (social gathering and holiday travel) factors might contribute to the COVID-19 epidemic outbreak and spread in theory. They identified a cold, dry winter to be a common environmental condition conducive for SARS virus infection to human beings [32]. In another study, Pirouz et al. (2020) [33] demonstrated that the relative humidity, maximum daily temperature, and average temperature had the highest impact statistically at the provincial level on confirmed cases of COVID-19. Liu et al. (2020) [34] explored the associations between COVID-19 case counts and meteorological factors in 30 provincial capital cities of China, finding that meteorological factors played an independent role in the COVID-19 transmission after controlling population migration, and that local weather conditions with low temperature, mild diurnal temperature range, and low humidity likely favored the transmission. Tosepu et al. (2020) [35] showed that, among the components of the weather, only temperature average was significantly correlated with the COVID-19 pandemic in Jakarta, Indonesia. Most recently, You et al. (2020) [36] demonstrated that the COVID-19 morbidity rate in Wuhan on 22 February 2020 was positively associated with the population density, construction land area proportion, value-added of tertiary industry per unit of land area, total retail sales of consumer goods per unit of land area, public green space density, aged population density and negatively associated with the average building scale, gross domestic production (GDP) per unit of land area, and hospital density. In summary, some natural, social, and economic variables such as temperature, relative humidity, population, and GDP, etc., were previously found to be significantly related to the COVID-19 epidemic mostly on a relatively large (big city or province) scale. Consequently, we hypothesized that influencing factors of the COVID-19 epidemic should have significant associations with these three kinds of variables on multiple scales.

The COVID-19 epidemic has been so far a pandemic, posing a severe threat to public health and social and economic development. Most recently, Liu et al. (2020) [37] investigated the spatial and temporal characteristics of nighttime light (NTL) radiance and air quality index (AQI) before and during the pandemic in mainland China and concluded that the outbreak and spread of COVID-19 had a crucial impact on people’s daily lives, and activity ranges through the increased implementation of lockdown and quarantine policies. However, there is still a lack of research on spatial statistical characteristics and influencing factors of the COVID-19 epidemic, especially from the prefecture level and county level scales, which could play a critical role in understanding the spatial evolution mechanism and in policymaking for prevention and control of the COVID-19 epidemic. It is imperative to research these topics to detect significant hotspots and clusters on relatively fine scales, such as the prefecture and county levels, and to help identify high-risk areas affected severely by COVID-19 for better prevention and control of the COVID-19 epidemic in practice. Hubei province had been the epicenter tragically affected by the COVID-19 epidemic. This lack of research had caused insufficient understandings of the epidemic and specific difficulties in the deployment of disease prevention and control materials as well as in the provision of precision medical support services at the initial outbreak in Hubei province.

Therefore, this study attempts to investigate the time-series spatial autocorrelation patterns and natural, social, and economic factors influencing the COVID-19 epidemic in Hubei province systematically from both prefecture and county levels using ArcGIS (Environmental Systems Research Institute, Inc., California, USA) spatial statistics and Spearman’s rank correlation methods. The aims of the study are to (i) enrich research on the epidemic temporal and spatial evolution and (ii) provide beneficial information for scientific prevention and control of COVID-19 epidemics.

To the best of our knowledge, this study was one of the first to investigate the spatial statistics and influencing factors of the COVID-19 epidemic on multiple scales. Four scientific contributions of this paper can be condensed as follows. First, a new, multiple-scale, and time-series geodatabase of Hubei province was constructed for the COVID-19 epidemic study, offering a finer spatial scale than that in the previous studies. Second, the global and local spatial autocorrelation methods were combinedly adopted in the detection of the areas of hotspots, clusters, and outliers for identification of the high-risk areas of the COVID-19 epidemic on multiple scales. Third, natural, social, and economic indicators were comprehensively considered to study the potential influencing factors at the prefecture and county levels of the COVID-19 epidemic. Lastly, except for the regular population indicators, the Baidu migration index (BMI) was used in the COVID-19 epidemic study as an alternative indicator signifying a population mobility factor.

## 2. Materials and Methods

### 2.1. Study Area

**Figure 1:**
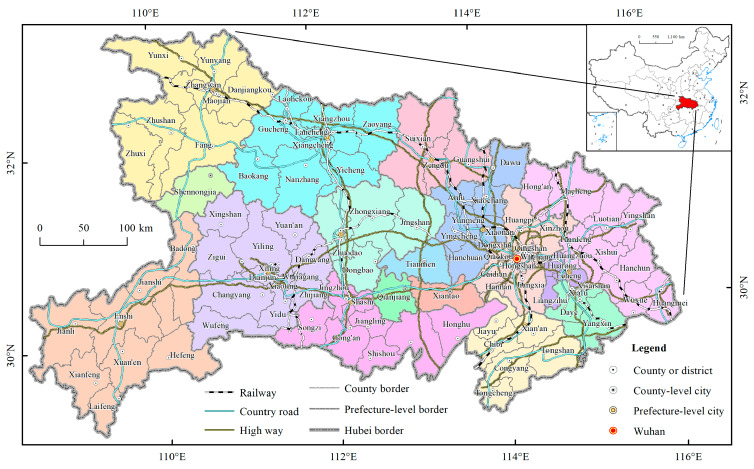
Map of the study area (location, administration, and transportation).

Hubei province is located in central China (108°21′42″ E~116°07′50″ E, 29°01′53” N~33°6’47″ N) (Figure 1). The province’s land area is 18.59 × 10^4^ km^2^, accounting for 1.94% of the country’s total land area. The terrain is mountainous in the east, west, and north and low in the central region, with an incomplete basin opening slightly to the south. The most area within the province has a subtropical monsoon humid climate, with an average annual temperature of 15 °C to 17 °C and average annual precipitation of 800 mm to 1600 mm. At present, Hubei province has 12 prefecture cities (Wuhan, Huangshi, Shiyan, Yichang, Xiangyang, Ezhou, Jingmen, Xiaogan, Jingzhou, Huanggang, Xianning, Suizhou), one autonomous prefecture (Enshi Tujia and Miao Autonomous Prefecture), one forest prefecture (Shennongjia forest prefecture), and three county level cities (Xiantao, Qianjiang, and Tianmen) directly under the provincial government, collectively referred to as 17 prefecture cities in the following analyses. The county level administrative units in Hubei province include 39 municipal districts, 22 county level cities (excluding the three cities directly under the provincial government), 36 counties, and two autonomous counties (Figure 1). As of the end of 2018, the province’s total resident population was 59 million, with an urbanization rate of 60.3%. The total road distance was 2.75 × 10^5^ km, of which high-grade highways accounted for 3.56 × 10^4^ km. The total passenger traffic was 9.87 × 10^8^ and the total GDP was 3.94 × 10^12^ RMB yuan with a growth ratio of 7.8%.

Wuhan city, the capital of Hubei province, is located in the eastern part of the Jianghan Plain and the middle reach of the Yangtze River. It is known as “the thoroughfare of nine provinces” due to its national comprehensive transportation hub. The land area of Wuhan city is 8.57 × 10^3^ km^2^. As of the end of 2019, the registered population and resident population in Wuhan city was 9.08 × 10^6^ and 14.18 × 10^6^, respectively. As of the end of 2018, there were 6.34 × 10^3^ health institutions, 9.59 × 10^4^ beds, 7.48 hospital beds per thousand people, 10.67 × 10^4^ health care personnel, and 3.42 doctors per thousand people in Wuhan city. The reported incidence rate of class A and B infectious diseases was 18 per ten thousand people for the year 2018.

### 2.2. Data Sources

Five kinds of datasets from different sources were used in this study, which included COVID-19 data, Baidu migration index data, social and economic statistical data, terrain (DEM), and geographic base map data as well.

First, the cumulative and daily new confirmed COVID-19 cases (23 January to 18 February 2020) for the 17 prefecture cities in Hubei province were obtained manually from the official website of the Hubei Provincial Health Committee. They were compiled to a dataset of prefecture level confirmed COVID-19 cases in Hubei province. Except for the districts and cities under the jurisdiction of Wuhan city, whose county level COVID-19 epidemic data were not reported publicly during the study period, the COVID-19 epidemic data (23 January to 18 February 2020) for the other counties (districts, cities) in Hubei province were obtained by hand from the official websites/newspapers/social media accounts of the prefecture governments or their affiliated health committees, and were compiled to a dataset of the county level confirmed COVID-19 cases. Additionally, a dataset for the daily new confirmed COVID-19 cases (DCC, 19 January to 18 February 2020) at the prefecture level in China was collected and provided by the Meishu Class news agency, *The Paper*. This dataset from *The Paper* was compiled to two sub-datasets, i.e., the provincial level and prefecture level datasets of cumulative and daily new confirmed COVID-19 cases nationwide in China. As a result, four kinds of COVID-19 epidemic datasets from different sources were collected and preprocessed according to three spatial statistical scales, i.e., the provincial, prefecture, and county levels.

Second, the prefecture level data of the Baidu migration index (BMI) were obtained from the Baidu Smart Eye Map (Baidu, Inc., Beijing, China). BMI represents a relative flow of population migration from a city to another in percentage, which can be applied in comparison to population migration at the same level of prefecture cities or provinces at present. We collected the data from Wuhan city and the other cities in Hubei province except for Shennongjia forest prefecture for the 16 days from 17 January to 1 February 2020, a period of massively large population movements and gathering for the Chinese Lunar New Year holidays traditionally. The Baidu Smart Eye Map does not provide the county level data of BMI, resulting in a lack of county level Spearman’s rank correlation between BMI and the number of CCC in this paper.

Third, the statistical data for the land area (LA), population density (PD), registered population (RGP), resident population (RSP), regional gross domestic product (GDP), and total retail sales of consumer goods (TRS) at the prefecture and county levels in Hubei province were obtained from the 2019 Statistical Yearbook of Hubei and the 2018 statistical yearbooks of the relevant prefecture cities in Hubei province, respectively. These data include only the yearly statistical figures in 2018 (a few in 2017).

Forth, the terrain (DEM, Digital Elevation Model) raster data of Hubei province with 90 m spatial resolution were downloaded from the Resource and Environmental Science Data Center of the Chinese Academy of Sciences (http://www.resdc.cn). The DEM data were derived from the SRTM (Shuttle Radar Topography Mission) data of the US space shuttle Endeavour in 2003. Zonal statistics in ArcGIS 10.7 were adopted to achieve four parameters, i.e., minimum elevation (MINE), maximum elevation (MAXE), mean elevation (MNE), and range of elevation (RAE) for each prefecture city and each county (or district) in Hubei province.

Fifth, the provincial level and prefecture level geographic base maps of China (2015) in the ArcGIS shapefile format were acquired from the Resource and Environmental Science Data Center of the Chinese Academy of Sciences. We updated them according to the Tianditu website, a national online platform for common geospatial information services in China. The county level geographic base map of China (1999) in the ArcGIS shapefile format was acquired from the website of the former National Bureau of Surveying and Mapping. The prefecture level and county level administrative maps of Hubei province were clipped from the relevant maps of China mentioned above with ArcGIS 10.7. Some county level borders in Hubei province were edited and updated with ArcGIS 10.7 according to the 2018 standard maps of the related prefecture cities in Hubei province, downloaded from the Hubei Natural Resources Department.

All of these data were double-checked and integrated carefully into an ArcGIS file Geodatabase for the COVID-19 epidemic study with a focus on Hubei province, of which the spatial feature data were linked with the attribute data by a common field, the geocode of an administrative area. It is worth noting that the time of the first confirmed case reported publicly varied from place to place, particularly for the county level data. Consequently, the periods of our studies for different scales were also various.

### 2.3. Research Methods

Two kinds of statistical approaches were used in the current study, of which the significant confidence levels were both 95% for the statistical tests.

Firstly, spatial autocorrelation technology was used to test whether the confirmed COVID-19 cases at the provincial, prefecture and county levels had significant global or local spatial autocorrelation characteristics, which was achieved by using Spatial Statistics and ModelBuilder Tools of ArcGIS 10.7. It has been widely used to study the spatial distribution of population [38,39], regional economic pattern [40], the epidemic situation [27,30,31,41,42,43], and urban thermal environment [44], and so forth. The spatial autocorrelation measures spatial autocorrelation based on both feature locations and feature values simultaneously. It can be divided into two methods: global spatial autocorrelation and local spatial autocorrelation. The Global Moran’s I index developed by Patrick A. P. Moran (1950) [45] is often used to measure the global spatial correlation, which is defined as Equation (1).

(1) $$I=\frac{n\sum_{i=1}^{n}\sum_{j=1}^{n}w_{ij}\left(x_{i}-\bar{x}\right)\left(x_{j}-\bar{x}\right)}{\sum_{i=1}^{n}{\left(x_{i}-\bar{x}\right)}^{2}\left(\sum_{i=1}^{n}\sum_{j=1}^{n}w_{ij}\right)},$$

where *n* is the total number of spatial units, *x* is an attribute of interest from a spatial unit, $\bar{x}$ is the mean of *x*, *w_ij_* is an element of the spatial weight matrix used to quantify the spatial relationship between spatial unit *i* and *j* (*I* ≠ *j*), and I denotes the global Moran’s I index. The value range of Moran’s I index is [–1, 1].

If Moran’s I index is statistically positive, there is a positive correlation in the spatial distribution, indicating a spatial clustering effect; otherwise, a negative spatial correlation exists. If Moran’s I index is zero, there is a random distribution. Statistical Z-score and *p*-value must be applied to determine statistical significance together with Moran’s I index. In this study, the global spatial autocorrelation was used to detect spatial characteristics of the confirmed COVID-19 cases and to analyze the overall spatial correlation within the entire study area.

Local spatial autocorrelation is commonly characterized by local Moran’s I and Getis-Ord G_i_*. Local Moran’s I is the decomposition of global Moran’s I into various sub-regional units, also known as Local Indicators of Spatial Association (LISA) [46]. In this study, cluster and outlier analysis (Anselin Local Moran’s I, ALMI) [46] of ArcGIS was used to detect the local spatial autocorrelation characteristics of the COVID-19 cases and identify the areas with significant-high/low clusters or outliers. Additionally, hot spot analysis (Getis-Ord G_i_*) of ArcGIS was used to identify the spatial association between hotspots and cold spots of COVID-19 cases with a statistical significance [47] on different spatial scales.

Secondly, Spearman’s rank correlation of SPSS 22 was used to examine relationships between the confirmed COVID-19 cases and the natural, population, and economic factors at the prefecture and county levels. The descriptive statistical results showed that the COVID-19 epidemic and relevant data do not meet the prerequisites of Pearson correlation analysis due to their non-Gaussian normal distributions, spatial autocorrelations, and possible nonlinear relationships. Generally, Spearman’s rank correlation is a suitable nonparametric estimator to estimate the correlation between two variables whose statistical distributions are unknown or non-Gaussian, and the relationship between these variables does not need to be linear [48]. It is commonly measured by the Spearman’s rank correlation coefficient, *ρ*, which is formulated as Equation (2).

(2) $$\rho =\frac{\sum_{i=1}^{n}\left(x_{i}-\bar{x}\right)\left(y_{i}-\bar{y}\right)}{\sqrt{\sum_{i=1}^{n}{\left(x_{i}-\bar{x}\right)}^{2}}\sqrt{\sum_{i=1}^{n}{\left(y_{i}-\bar{y}\right)}^{2}}}=\frac{cov\left(x,y\right)}{S_{x}S_{y}}\;,$$

where *n* is the total number of samples, *x_i_* and *y_i_* is the rank of the variable *X_i_* and *Y_i_*, respectively, $\bar{x}$ and $\bar{y}$ is the mean rank of *X* and *Y*, respectively, *cov*(*x,y*) is the covariance of *x* and *y*, *S_x_* and *S_y_* is the product of their standard deviations, respectively, and *ρ* denotes the Spearman’s rank correlation coefficient.

This coefficient varies between −1 and +1, with +1 (−1) signifying a perfect positive (negative) correlation between the two variables. The greater the absolute value of Spearman’s *ρ* is, the stronger the relationship between the two variables is. Like Pearson’s coefficient, the absolute value of Spearman’s *ρ* ranging 0.8~1 indicates a correlation is extremely strong, 0.6~0.8, strong, 0.4~0.6, moderate, 0.2~0.4 weak, and 0~0.2, no correlation. The Spearman’s rank correlation has been used in recent studies on hemorrhagic fever with renal syndrome [44], urban heat island [48], ozone pollution [49], COVID-19 [35], and so on.

## 3. Results

### 3.1. Spatial Statistics of the COVID-19 Epidemic

This section analyzed the time-series variation characteristics of global spatial autocorrelation and local spatial autocorrelation of the COVID-19 epidemic in Hubei province from three scales: the provincial, prefecture, and county levels by using Global Moran’s I, cluster and outlier analysis, and hot spot analysis (Getis-Ord G_i_*) tools of ArcGIS 10.7. Primarily, this study used 34 spatial units nationwide for the provincial level spatial statistics analysis from 19 January to 18 February 2020, 359 spatial units nationwide for the prefecture level spatial statistics analysis from 19 January to 18 February 2020, 103 spatial units province-wide, including 38 counties, 39 districts, 22 county level cities, three prefecture cities, and Shennongjia forest prefecture for the county level spatial statistics analysis mainly from 30 January to 18 February 2020.

#### 3.1.1. Spatial Autocorrelations of the Provincial COVID-19 Outbreaks Nationwide

The results indicated that from 19 January to 18 February 2020, there was no significant global spatial autocorrelation (*p* > 0.05, *Z* < 1.96) for the number of CCC at the provincial (municipal and autonomous regions) level nationwide.

The Anselin Local Moran’s I (ALMI) analysis results (Figures omitted) showed that from 19 January to 18 February 2020, Hubei province was the only significant high-low outlier area (the 95% confidence) of the number of CCC in China nationwide during these 31 days while some surrounding provinces (for instance, Henan province, Hunan province, and Chongqing city) were the significant low-high outlier areas.

The inverse distance-based hot spot analysis (Getis-Ord G_i_*) results (Figures omitted) showed that Hubei province was the only extremely significant hotspot area (the 99% confidence) for the number of CCC at the provincial level in China nationwide during these 31 days from 19 January to 18 February 2020.

The results of the local spatial statistics revealed that Hubei province had always been the most severe COVID-19 epidemic area with a significant high risk at the provincial level in China during the initial period of the epidemic.

#### 3.1.2. Spatial Autocorrelations of the Prefecture Level COVID-19 Outbreaks Nationwide

**Figure 2:**
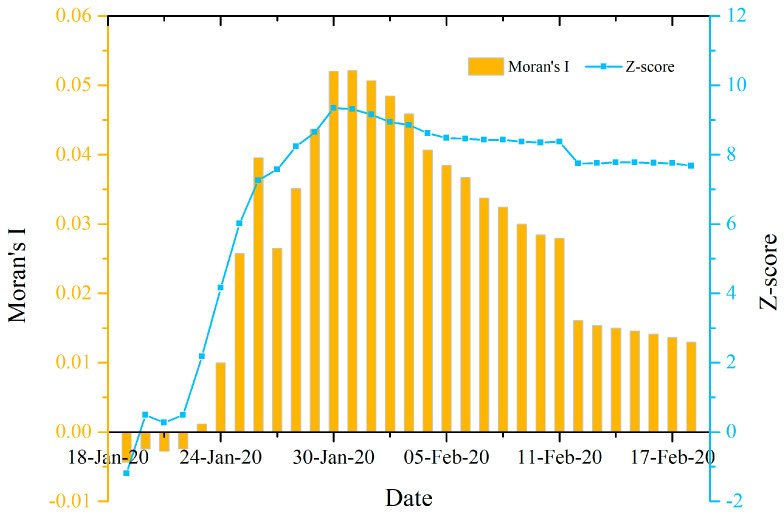
Global spatial autocorrelation analysis results of the number of the cumulative confirmed COVID-19 cases at the prefecture level nationwide in China from 19 January to 18 February 2020.

Figure 2 shows that, from 19 January to 22 January 2020, there was no significant global spatial autocorrelation (*p* > 0.05, Z < 1.96) for the prefecture level CCC nationwide, which may be due to the insufficient amount of the prefecture cities reporting the number of CCC in China during this period. On 23 January 2020, there was a significant global spatial autocorrelation (*p* < 0.05, Z > 1.96) for the prefecture level CCC nationwide. From 24 January to 18 February 2020, the prefecture level cumulative COVID-19 confirmed cases demonstrated an extremely significant global spatial autocorrelation (*p* < 0.0001, Z > 2.58), indicating a very significant concentrated distribution for the nationwide prefecture level COVID-19 cases. The trend of Moran’s I index in Figure 2 displayed two phases of variation characteristics: an initial increase and then a decrease around 30 January 2020, indicating that this date could be a turning point for the global spatial autocorrelation (Moran’s I index) of the number of CCC at the prefecture level nationwide. It is noted that the absolute values of Moran’s I index are all less than 0.06 during the study period, indicating an extremely weak spatial autocorrelation.

**Figure 3:**
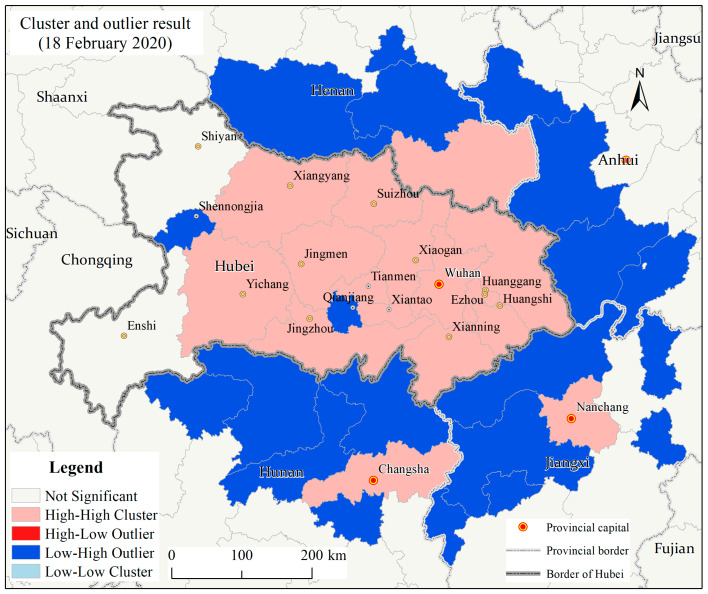
Cluster and outlier analysis result of the number of the cumulative confirmed COVID-19 cases at the prefecture level in Hubei province and its surrounding areas on 18 February 2020.

The Anselin Local Moran’s I (ALMI) results revealed that from 19 January to 18 February 2020, most prefecture cities in Hubei province were the significant high-high cluster areas (the 95% confidence) for the prefecture level CCC while some prefecture cities neighboring Hubei province were low-high outlier areas (the 95% confidence) nationwide. Figure 3 shows that, on 18 February 2020, there were 13 prefecture cities, namely Wuhan, Xiaogan, Huanggang, Ezhou, Huangshi, Xianning, Suizhou, Xiangyang, Jingmen, Yichang, Jingzhou, Tianmen, and Xiantao in Hubei province, being all significant high-high cluster areas (the 95% confidence) for the number of CCC. Additionally, their surrounding cities, Xinyang in Henan province, Changsha in Hunan province, and Nanchang in Jiangxi province were the significant high-high cluster areas (the 95% confidence) for the number of CCC. In contrast, some areas closely adjacent to these prefecture cities were the significant low-high outliers (the 95% confidence).

**Figure 4:**
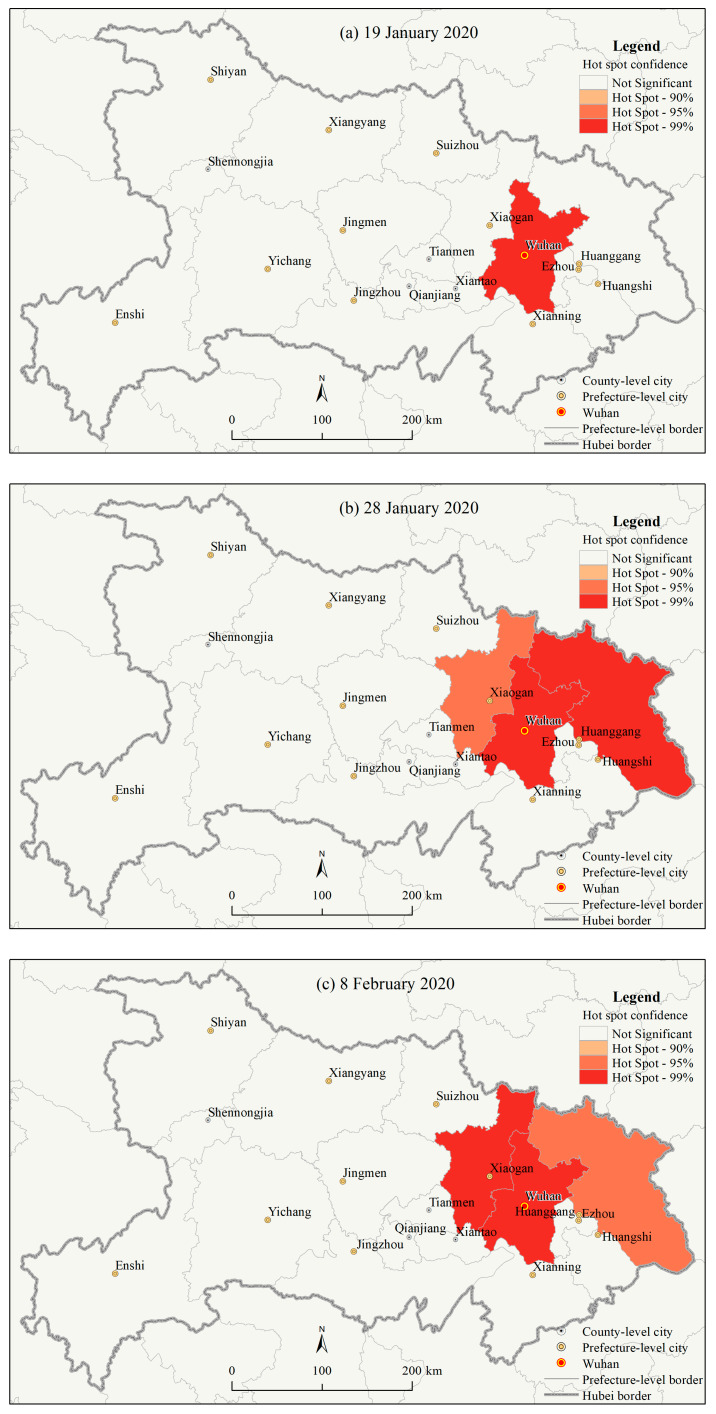

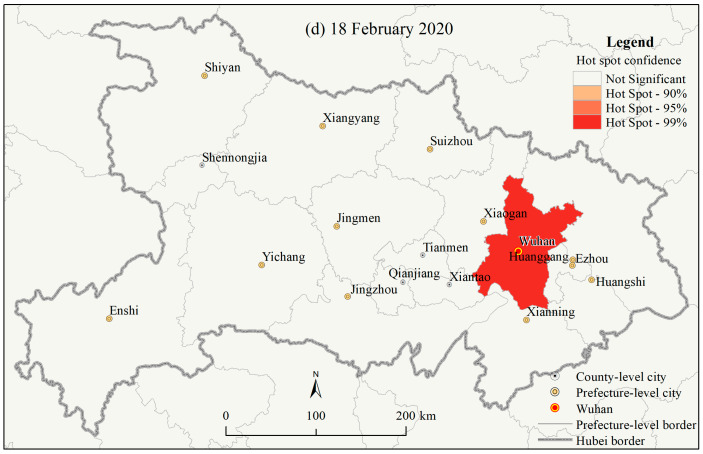
Hotspot (Getis-Ord G_i_*) analysis results of the number of cumulative confirmed COVID-19 cases at the prefecture level in Hubei province on (**a**) 19 January 2020, (**b**) 28 January 2020, (**c**) 8 February 2020, and (**d**) 18 February 2020.

The inverse distance-based hot spot analysis (Getis-Ord G_i_*) results (Figure 4, Figures omitted for the other dates) indicated that in Hubei province, from 19 January to 18 February 2020, Wuhan city was the extremely significant hotspot area (the 99% confidence, Figure 4a–d) for the number of CCC at the prefecture level during all the period. On 24 January 2020, Huanggang city became a significant hotspot area (the 95% confidence) due to the rapid increase in the number of DCC. It became further an extremely significant hotspot area (the 99% confidence) on 25 January. Then it returned to a significant hotspot area (the 95% confidence, Figure 4c) after 8 February. Due to an odd increase in the number of DCC in Wuhan city after 12 February 2020, Huanggang city became a nonsignificant area compared to Wuhan city. On 28 January 2020, the rapid increase in the number of CCC in Xiaogan city made it a significant hotspot area (the 95% confidence, Figure 4b). It further became an extremely significant hotspot area (the 99% confidence) on 29 January 2020.

Moreover, it again became a significant hotspot area (the 95% confidence) on 11 February 2020. For the same reason, after 12 February 2020, Xiaogan city became a nonsignificant area compared to Wuhan city. Although the other prefecture cities in Hubei province also had a large number of CCC compared to the other domestic cities in China, they were not significant hotspots due to the oddly high CCC in Wuhan city. The results suggested that Wuhan city was the prefecture city with the most severe and intensive COVID-19 epidemic in Hubei province over the study period and that Huanggang city and Xiaogan city were also among the prefecture cities with the most severe and intensive COVID-19 epidemic in Hubei province for some time. In all, it was found from the prefecture level that Wuhan city had been the most high-risk area, followed by Huanggang city and Xiaogan city, in Hubei province during the initial outbreak and spread of the epidemic.

#### 3.1.3. Spatial Autocorrelations of the County Level COVID-19 Outbreaks in Hubei Province

Because the county level COVID-19 epidemic data were not reported publicly in Wuhan city during the study period, we collected the county level COVID-19 epidemic data solely for the other prefecture cities except Wuhan city in Hubei province. To exclude the statistical instability due to the exceptionally high cumulative cases as a whole in Wuhan city, the weighted average method by the county level RSP was used to divided CCC into the corresponding districts/cities in Wuhan city to perform spatial autocorrelation analysis. Due to data availability, we selected the county level COVID-19 data from 30 January to 18 February 2020, for the study.

**Figure 5:**
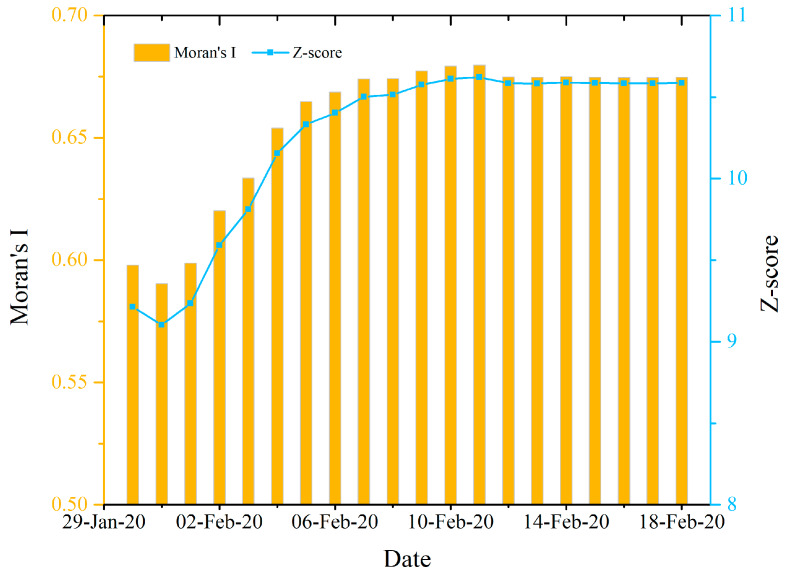
Global spatial autocorrelation analysis results for the cumulative confirmed COVID-19 cases at the county level in Hubei province from 30 January to 18 February 2020.

The global spatial autocorrelation results (Figure 5) illustrated that from 30 January to 18 February 2020, the number of CCC in Hubei province at the county level had an extremely significant spatial autocorrelation (*p* < 0.0001, *Z* > 2.58) and that the global Moran’s I index and Z-score increased since 31 January. It indicated that the county level COVID-19 epidemic in Hubei province had a very significant concentrated characteristic and that the spatial autocorrelation became increasingly intensive from 31 January to 18 February 2020. It is noted that the values of Global Moran’s I index are all positive and greater than 0.59, and the Z-score values are all greater than 9.0 during the study period, indicating a significant and strong global spatial autocorrelation. In other words, the county level epidemic in Hubei province had a significant and strong spatial dependence between the neighboring counties during the study period.

**Figure 6:**
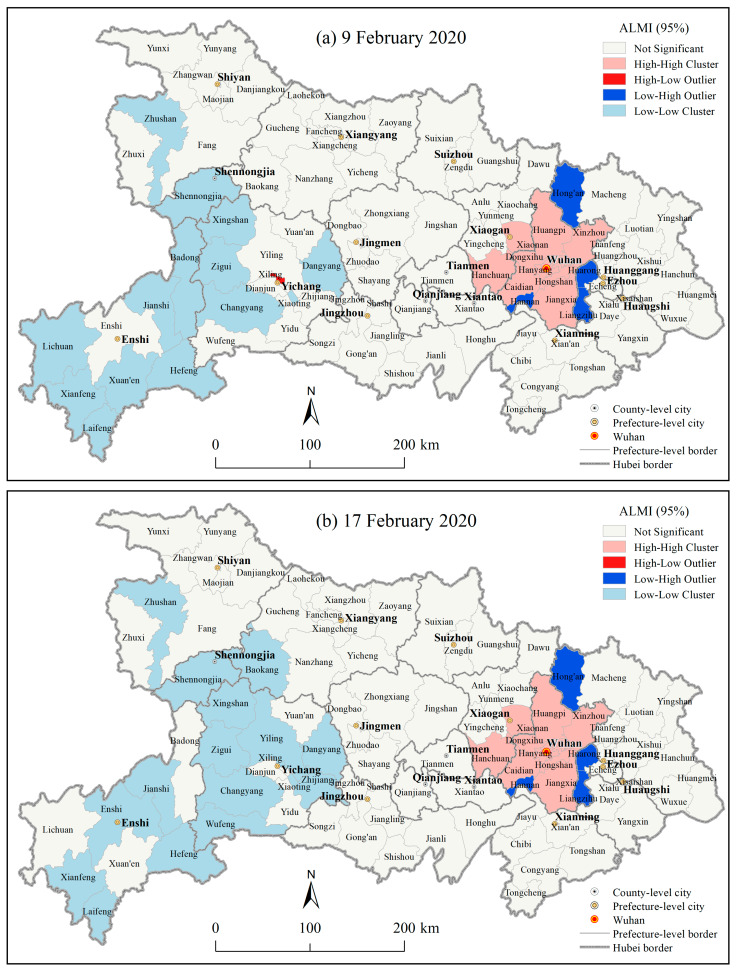
ALMI results of the cumulative confirmed COVID-19 cases at the county level in Hubei province on (**a**) 9 February 2020, and (**b**) 17 February 2020.

The Anselin Local Moran’s I (ALMI) results (Figure 6, Figures omitted for the other dates) demonstrated that from 30 January to 18 February 2020, there were significant clusters and outlier areas (*p* < 0.05) regarding the number of CCC at the county level in Hubei province. Wuhan’s 12 districts (excluding Hannan district) and its neighboring Xiaonan district and Hanchuan city in Xiaogan city were the significant high-high cluster areas (Figure 6, *p* < 0.05). The Hannan district in Wuhan city and its neighborhood Hong’an county in Huanggang city, as well as Huarong district and Liangzihu district in Ezhou city were the significant low-high outlier areas (*p* < 0.05), and most county level units in southwest Hubei were the significant low-low cluster areas (*p* < 0.05). It indicated that the county level COVID-19 epidemic in Wuhan city was significantly severe (*p* < 0.05) during this period. Notably, Xiling district in Yichang city showed a significant high-low outlier (*p* < 0.05), from 30 January to 9 February 2020 (Figure 6), indicating that the number of CCC in this county was significantly higher (*p* < 0.05) than its surroundings and causing Xiling district a significant high-risk area (*p* < 0.05) of the COVID-19 epidemic compared to its surrounding areas. From 10 February to 18 February 2020, this district was not a significant abnormal area, which indicated that the COVID-19 epidemic in the district had relatively alleviated during this period.

**Figure 7:**
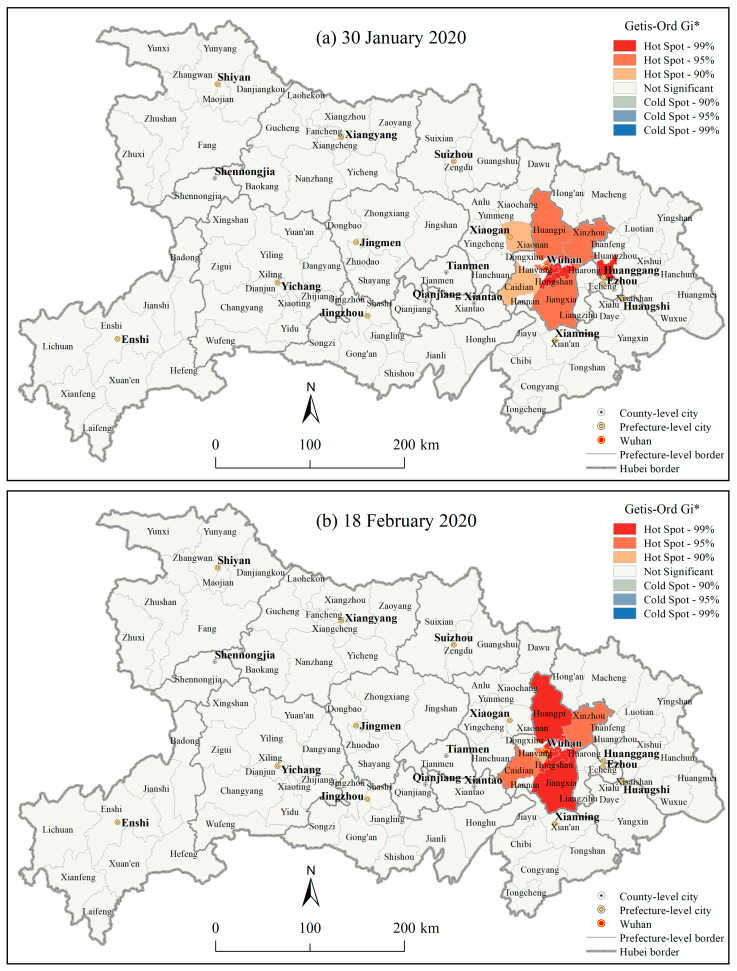
Hotspot analysis (Getis-Ord G_i_*) results of the cumulative confirmed COVID-19 cases at the county level in Hubei province on (**a**) 30 January 2020, and (**b**) 18 February 2020.

The inverse distance-based Hot Spot Analysis (Getis-Ord G_i_*) results (Figure 7, Figures omitted for the other dates) showed that from 30 January to 18 February 2020, the extremely significant hotspot areas (the 99% confidence) and significant hotspot areas (the 95% confidence) for the county level CCC in Hubei province were limitedly located in some urban areas such as Huangpi district and Jiangxia district in Wuhan city as well as its nearby Huangzhou district in Huangzhou city. There were some changes in the significant hotspot areas of CCC at the county level in Wuhan city, which are not described in detail here. Although the confirmed COVID-19 cases in the other counties in Hubei province were also relatively high, there were no significant hotspots observed because the confirmed COVID-19 cases among those districts in Wuhan city were too extremely high.

In short, the local spatial autocorrelation analysis results revealed that the districts in Wuhan city were the areas with the most severe COVID-19 epidemic at the county level in Hubei province, which could be seen as the significant high-risk areas.

### 3.2. Influencing Factors of the COVID-19 Epidemic

A study on influencing factors of the COVID-19 epidemic may help to provide an updated understanding of its spread mechanism and assist its prevention and control. As demonstrated above, the COVID-19 epidemic for CCC in Hubei province took on the evident characteristics of extremely concentrated outbreaks and significant spatial autocorrelations. However, the influencing factors of the COVID-19 epidemic regarding these characteristics remain unresolved. In order to provide helpful information for the risk factor identification and the prevention and control of the COVID-19 epidemic, this study explored the influencing factors of the prefecture level and county level COVID-19 spreads in Hubei province from natural, social, and economic aspects by using the Spearman’s rank correlation of SPSS 22. We intentionally chose eleven indicators, i.e., four elevation parameters (MINE, MAXE, MNE, and RAE), LA, four population indicators (PD, RGP, SP, and BMI), and two economic indicators (GDP and TRS) for the prefecture level Spearman’s rank correlation analysis and ten indicators for the county level Spearman’s rank correlation analysis except for BMI due to no access to the relevant data temporarily. Particularly, this study used 17 spatial units province-wide for the prefecture level correlation analysis from 23 January to 18 February 2020. For the county level correlation analysis, this study used 88 spatial units province-wide, excluding 13 districts/cities in Wuhan city, one district in Jingzhou city, and Shennongjia forest prefecture from 26 January to 18 February 2020.

#### 3.2.1. Influencing Factors of the Prefecture Level COVID-19 Outbreaks in Hubei Province

The Spearman’s rank correlation results (Table 1) demonstrated that the number of CCC at the prefecture level in Hubei province had a strong, positive, and extremely significant correlation (*p* < 0.01) with RGP from 23 January to 18 February 2020; had a positive, and extremely significant correlation (*p* < 0.01) with RSP on 23 January 2020, and from 25 January to 18 February 2020, respectively, of which the correlations were strong except for the extremely strong ones on 2 February and 4 February 2020; had a positive and extremely significant correlation (*p* < 0.01) with TRS from 25 January to 18 February 2020, respectively, of which the correlations were strong except for the extremely strong ones from 31 January to 4 February 2020, and 8 February 2020; had a positive and extremely significant correlation (*p* < 0.01) with GDP on 23 January 2020, and from 25 January to 18 February 2020, respectively, of which the correlations were strong except for the extremely strong ones from 29 January to 9 February 2020; had a strong, positive, and extremely significant correlation (*p* < 0.01) with BMI on 27 January 2020, and from 29 January to 18 February 2020, respectively, while had a nearly strong, positive and significant correlation (*p* < 0.05) with BMI on 23, 25, 26, and 28 January 2020, respectively; had a moderate, positive, and significant correlation (*p* < 0.05) with PD only from 13–18 February 2020; had a moderate, negative and significant correlation (*p* < 0.05) with MINE on 22 January 2020, from 25–27 January 2020, and from 2–18 February 2020, respectively; nevertheless, the correlation with MAXE, MNE, RAE, LA, and PD was not significant (*p* > 0.05) from 23 January to 18 February 2020, respectively.

The average number of CCC at the prefecture level in Hubei province from 23 January to 18 February 2020, was calculated for the correlation with the eleven indicators mentioned above in order to eliminate the instability of data and to validate the analysis results of time series. The results (Table 1) showed that this average value had a strong, positive, and extremely significant correlation (*p* < 0.01) with RGP, RSP, TRS, GDP, and BMI, respectively; had a moderate, negative, and significant correlation (*p* < 0.05) with MINE; in contrast, the correlation with MAXE, MNE, RAE, LA, and PD was not significant (*p* > 0.05), respectively.

The results suggested that social and economic development and population movements could have strong impacts on the COVID-19 epidemic spread. The result showed that the Spearman’s rank correlation coefficient between the number of CCC and RSP was all greater than that for RGP from 26 January to 18 February 2020, respectively. It suggested that the more population and the greater PD at the early outbreak stage, the greater the risk of infection of SARS-CoV-2 could be, and the severer the COVID-19 epidemic thus could be. Interestingly, the variation trend of the Spearman’s rank correlation coefficient for the number of CCC and GDP was entirely in agreement with that and TRS during all the study period, of which the coefficients both were around 0.70 or above, a strong correlation level, with the latter all greater than the former. This suggested that economic development and trade exchanges could positively contribute to the epidemic spread. It is worth pointing out that this does not mean that the more developed the economy is, the stronger the epidemic will be, but the bigger the infectious risk will be. To sum up, from the point of view of CCC at the prefecture level in Hubei province, population (PD, RSP, RGP, and BMI), economic development (GDP), and trade exchanges (TRS) could be the high-risk factors to the COVID-19 epidemic spread.

In addition to the correlation analysis for the number of CCC, we also analyzed the number of DCC. The Spearman’s rank correlation analysis results (Table S1) showed that there were relatively complex correlations between the number of DCC and the eleven indicators analyzed at the prefecture level in Hubei province, not consistent with those between the number of CCC and the corresponding indicators except for no significant correlation (*p* > 0.05) for LA, MAXE, MNE, and RAE. The number of DCC at the prefecture level in Hubei province had a strong, positive, and extremely significant correlation (*p* < 0.01) with TRS and GDP, respectively, from 25 January to 8 February 2020; that correlation with TRS was moderate, positive, and significant (*p* < 0.05) on 9 and 10 February 2020, and not significant (*p* > 0.05) from 11–18 February 2020; that correlation with GDP was moderate, positive, and significant (*p* < 0.05) from 9–11 February 2020, on 14 and 18 February 2020, and was nevertheless not significant (*p* > 0.05) on any other day. The number of DCC at the prefecture level in Hubei province had a strong, positive, and extremely significant correlation (*p* < 0.01) with RGP and RSP, respectively, from 25 January to 6 February 2020, and on 8 February 2020; had a moderate, positive, and significant correlation (*p* < 0.05) on 7 February 2020, and from 9–11 February 2020; had no significant correlation (*p* > 0.05) on any other day nevertheless.

However, the number of DCC at the prefecture level in Hubei province had no steadily significant correlation (*p* < 0.05) with PD, BMI, and MINE from 24 January to 18 February 2020 (Table S1). The correlation between the number of DCC and PD at the prefecture level in Hubei province were significant (*p* < 0.01 and *p* < 0.05) only for 12 discontinuous days. The correlation between the number of DCC and BMI at the prefecture level in Hubei province was complex. It was strong, positive, and extremely significant (*p* < 0.01) from 27–30 January, on 2 and 4 February, from 6–8 February, and on 14 February 2020; was positive and significant (*p* < 0.05) on 31 January and 1, 3, 5, 9, and 11 February 2020, and was not significant on any other day. The correlation between the number of DCC and MINE at the prefecture level in Hubei province was also complex, with a significance only for 11 discontinuous days.

The 26-days average number of DCC at the prefecture level in Hubei province from 24 January to 18 February 2020 had a strong, positive, and extremely significant correlations (*p* < 0.01) with RGP, RSP, TRS, GDP, and BMI, respectively. The correlation between this average value and PD was moderate, positive, and significant (*p* < 0.05). The correlation between this average value and MINE was moderate, negative, and significant (*p* < 0.05). However, the correlations with MAXE, MNE, RAE, and LA were not significant (*p* > 0.05) at the prefecture level in Hubei province.

#### 3.2.2. Influencing Factors of the County Level COVID-19 Outbreaks in Hubei Province

The Spearman’s rank correlation analysis results (Table 2) indicated that the number of CCC at the county level in Hubei province had a moderate, positive, and extremely significant correlation (*p* < 0.01) with PD from 26–30 January 2020, and had a strong, positive, and extremely significant correlation (*p* < 0.01) with PD on from 31 January to 18 February 2020, respectively; that had a moderate, positive, and extremely significant correlation (*p* < 0.01) with TRS from 26–28 January 2020, and had a strong, positive, and extremely significant correlation (*p* < 0.01) from 29 January to 18 February 2020, respectively; that had a weak, positive, and significant correlation (*p* < 0.05) on 26 January 2020, had a weak, positive, and extremely significant correlation (*p* < 0.01) with RGP on 27 January 2020, and had a moderate, positive, and extremely significant correlation from 28 January to 18 February 2020, respectively; that had a weak, positive and significant correlation (*p* < 0.05) with RSP on 26 January 2020, had a moderate, positive, and extremely significant correlation (*p* < 0.01) with RSP from 28 January to 1 February 2020, and had a strong, positive, and extremely significant correlation (*p* < 0.01) with RSP from 2 February to 18 February 2020, respectively; that had a weak, positive and significant correlation (*p* < 0.05) with GDP on 26 January 2020, had a weak, positive and extremely significant correlation (*p* < 0.01) with GDP on 27 January 2020, had a moderate, positive, and extremely significant correlation (*p* < 0.01) with GDP from 28–29 January, on 2 February 2020, and from 4 February to 18 February 2020, and had a strong, positive, and extremely significant correlation (*p* < 0.01) with GDP from 30 January to 1 February 2020 and on 3 February 2020, respectively; and that had a weak, negative and significant correlation (*p* < 0.05) with LA on 26 January 2020, and from 30 January to 1 February had no significant correlation from 27–29 January 2020, and had a weak, negative, and extremely significant correlation (*p* < 0.01) with LA from 2–18 February 2020, respectively.

Interestingly, the results (Table 2) showed that the number of CCC at the county level in Hubei province had a negative and significant correlation with all the four elevation parameters, i.e., MINE, MAXE, MNE, and RAE, respectively, from 26 January to 18 February 2020. It had a weak and significant correlation (*p* < 0.05) with MINE from 26–27 January 2020; had a weak and extremely significant correlation (*p* < 0.01) with MINE from 28–31 January 2020; had a moderate and extremely significant correlation (*p* < 0.01) with MINE from 2–18 February 2020. It had a moderate and extremely significant correlation (*p* < 0.01) with MAXE from 26 January to 3 February 2020; it had a strong and extremely significant correlation (*p* < 0.01) with MINE from 4–18 February 2020. It had a moderate and extremely significant correlation (*p* < 0.01) with MNE from 26 January to 1 February 2020; it had a strong and extremely significant correlation (*p* < 0.01) with MNE from 2–18 February 2020. Furthermore, it had a moderate and extremely significant correlation (*p* < 0.01) with RAE from 26 January to 5 February 2020; it had a strong and extremely significant correlation (*p* < 0.01) with RAE from 6–18 February 2020.

In order to ensure the reliability of data and to validate the analysis results of time series, the average of the number of CCC at the county level in Hubei province from 26 January to 18 February 2020, was calculated for the correlation with the ten indicators mentioned above. Table 2 shows that this average value had a strong, positive, and extremely significant correlation (*p* < 0.01) with PD, RSP, TRS, respectively; it had a moderate, positive, and extremely significant correlation (*p* < 0.01) with RGP and GDP, respectively; it had a weak, negative, and extremely significant correlation (*p* < 0.01) with LA; it had a moderate, negative, and extremely significant correlation (*p* < 0.01) with MINE; it had a strong, negative, and extremely significant correlation (*p* < 0.01) with MAXE, MNE, and RAE, respectively. It should be noted that the results were almost the same as those of time series.

The correlation analysis of DCC at the county level in Hubei province (Table S2) did not establish any stable correlation with the ten indicators analyzed during the period from 26 January to 18 February 2020. However, the average of DCC at the county level had a strong, positive, and extremely significant correlation (*p* < 0.01) with PD, RSP, RSP, and TRS, respectively; it had a moderate, positive, and extremely significant correlation (*p* < 0.01) with RGP and GDP, respectively; it had nevertheless a weak, negative, and extremely significant correlation (*p* < 0.01) with LA; it had a moderate, negative, and extremely significant correlation (*p* < 0.01) with MINE; it had a strong, negative, and extremely significant correlation (*p* < 0.01) with MAXE, MNE, and RAE, respectively.

## 4. Discussion

### 4.1. Geographic Risk Identification Based on the Spatial Statistics of the COVID-19 Epidemic

Knowledge mining of geospatial information can not only reveal the spatiotemporal spread and aggregation characteristics of infectious diseases, but also discover spatial risk factors that have an important influence on the spread of infectious diseases and identify hot spots with high transmission risks [27]. It is of great significance for the scientific prevention and control of an epidemic. Geographic and environmental risk identification of infectious diseases has drawn great attention from various fields of researchers [8,27,31,41,50,51,52]. Spatial statistics analysis has been used as a tool to identify geographic and environmental risks of epidemics such as SARS 2003 [25,27] and COVID-19 [30]. Cao et al. (2008) revealed that high-high cluster areas signifying the high-risk areas were mainly in the center of Guangzhou city, which has a high population density, is economically active, and accommodates a well-developed traffic net [27]. The study of Kang et al. revealed a significant spatial association of COVID-19 infections in China from around 22 January 2020 [30], which was consistent with the results of our present study. As shown in Section 3.1, the local spatial autocorrelation of the COVID-19 epidemic can help to effectively detect the significant cluster/outlier areas and also the significant hotspot areas at different spatial levels in Hubei province. Based on this, it can be inferred that Hubei province, Wuhan city, and most of the districts in Wuhan city had been the most high-risk areas at the provincial, prefecture, and county levels, respectively, during the initial spread of the epidemic.

Figure 2 and Figure 5 show the results of global spatial autocorrelation of the COVID-19 epidemic at the prefecture and county levels in Hubei province, respectively. Comparing these two figures, it can be found that the global spatial autocorrelation of the county level CCC in Hubei province was significantly higher than that of the prefecture level CCC and that their autocorrelation variation trends were very different from 31 January to 18 February 2020. Moreover, the global spatial autocorrelation of the prefecture level CCC had a downward trend during the period while that of the county level CCC had an upward trend from 31 January to 11 February 2020, and sustained a steady status from 12–18 February 2020. As stated above, there was no significant global spatial autocorrelation for CCC at the provincial level nationwide. Therefore, it can be inferred that the spatial scale could have a significant effect on the global spatial autocorrelation of the COVID-19 epidemic. In other words, the global spatial autocorrelation of the COVID-19 epidemic could have a significant dependence on the spatial scale. The finer the spatial scale, the closer the spatial unit would be, and thus the stronger the global spatial autocorrelation of the COVID-19 epidemic would be. This agrees with the Tobler’s first law of geography. This finding indicated that the closer to the high-risk area, the greater the risk was, especially on the county level scale. This finding could benefit governments at different levels to take effective and classified prevention and control measures.

### 4.2. Potential Risk Factors of the COVID-19 Spread

In general, the factors that affect an epidemic outbreak and spread mainly occur through the influences on the source of infection, the route of transmission, and the susceptible population. These factors have close relationships with individuals microcosmically while with the natural environment and socio-economic development macroscopically. On the whole, it is believed that the COVID-19 epidemic spread could be closely related to certain natural, social, and economic factors as well as to prevention and control policies controlling sources of the outbreak and cutting off routes of transmission. Recently, researchers proposed that some meteorological parameters such as temperature and humidity were potential environmental factors influencing the outbreak of COVID-19 [32]. Most lately, scholars showed that the COVID-19 morbidity rate in Wuhan on 22 February 2020 was highly associated with some social and economic indicators [36]. Previous studies analyzed the influencing factors of the COVID-19 epidemic only on one spatial scale.

However, this study expounded the influencing factors of CCC from all three aspects of nature, society, and economy at both prefecture and county levels in Hubei province. Furthermore, this study analyzed different indicators from previous works for potential risk factors of the COVID-19 spread. In this paper, land area and four terrain parameters (MINE, MAXE, MNE, and RAE) were taken as natural indicators, while GDP and TRS were two economic indicators, and PD, RGP, RSP, and BMI were four key social indicators. More specifically, BMI signified the population mobility. Our results showed that most of these three kinds of indicators were highly related to the COVID-19 epidemic to some extent. This was in line with the study of You et al. (2020) [36]. In nature, most of these factors need to work through the population. Therefore, the spread of the COVID-19 epidemic would have a high correlation with the population mobility. The more developed the society and economy, the strong the population mobility and economic exchange could be, and thus the severer the epidemic risk could be at the initial outbreak stage. It was surprising that the PD factor played different roles in influencing the epidemic spread at the prefecture and county levels. This was significantly associated with the number of CCC at the county level, but not at the prefecture level, indicating a spatial scale effect to some extent. Its reason needs further in-depth study.

**Figure 8:**
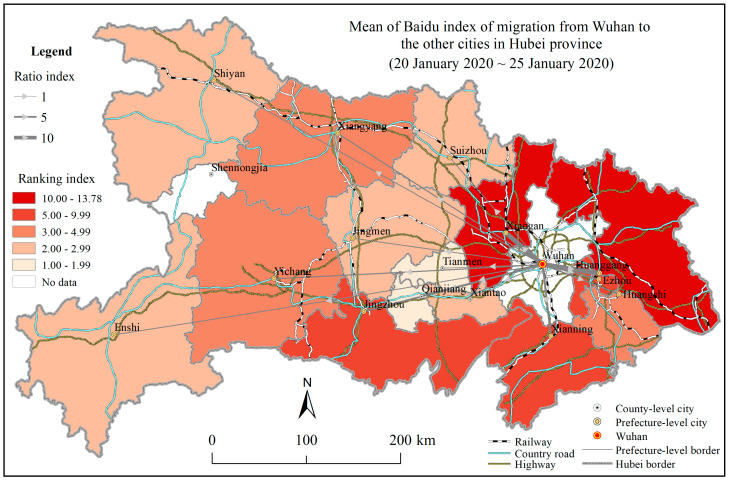
Thematic map of the mean of Baidu migration index from Wuhan to the other cities in Hubei province from 20 January to 25 January 2020.

In particular, BMI can be taken as a quantitative indicator for the population migration and mobility from one place to another and possible for the social gathering and holiday travel. As expected, the number of CCC had an extremely strong dependence on BMI, indicating that population mobility would significantly increase the risk of the COVID-19 spread at the early epidemic stage. Figure 8 shows that BMIs for Wuhan to Xiaogan and Huanggang were largely higher than that for Wuhan to any of the other cities in Hubei province, which was consistent with the current statistical results of the COVID-19 epidemic in Xiaogan and Huanggang compared to that experienced in the other cities in Hubei province. Wuhan city, as the capital city of Hubei province and the core city of China’s central urban agglomeration, had a strong impact on its surrounding cities [53]. Its connections through the traffic and economic ties with Xiaogan, Huanggang, Jingzhou, and Xianning were higher than that with the other cities in Huber province [54,55].

Consequently, there could be huge population movements and commercial communications between Wuhan and these cities in Hubei province before the implementation of strict quarantine policy, increasing the infectious chance of the COVID-19 spread threat. This might be the reason that the number of CCC at the prefecture level in Hubei province had an extremely significant positive correlation with BMI. Therefore, BMI could be a potential risk indicator for the COVID-19 spread, like the other social and economic indicators.

Moreover, comparing Table 1 and Table 2, it can be found that the correlation coefficients between the number of CCC and RSP at the prefecture and county levels in Hubei province were larger than that for RGP, indicating that RSP may have had a more critical impact on the spread of COVID-19. The correlation coefficient between the number of CCC at both prefecture and county levels in Hubei province and TRS was higher than that for GDP. It should also be noted that the correlation coefficients for the number of CCC and various indicators at the prefecture level in Hubei province were obviously higher than those for the corresponding indicators at the county level. The findings indicated that the indicators of RSP and TRS could be more suitable ones as the potential risk factors impacting on the spread of the COVID-19 epidemic than did RGP and GDP, especially on the prefecture level scale. The correlation with PD and LA changed contrarily on the two scales. Thus, the reasons and mechanisms of the spatial scale effect are worthy of further study.

### 4.3. Limitations

Because the COVID-19 data analyzed were collected from the different public sources of the 17 prefecture cities and more than 100 county level administrative districts in Hubei province and had various kinds of unstandardized and unstructured formats, these data had to be manually processed and integrated in a time-consuming and labor-intensive manner, especially for the county level data in Hubei province. Moreover, it was recently reported that the official COVID-19 epidemic data might not include asymptomatic cases, which would impact the accuracy of data analyzed. Furthermore, multicollinearity, spatial heterogeneity, and temporal inconsistency between the socioeconomic data and the epidemic data very likely existed [36]. These uncertainties were not considered in this paper. Therefore, the following notable limitations might occur and should be addressed in the present study.

First, the mechanism controlling the spatiotemporal patterns and the COVID-19 spread on different scales has not been resolved. It is an interdisciplinary and complex problem in theory. Our results showed that CCC at the provincial, prefecture and county levels had significant local spatial autocorrelation and clustering characteristics, but there was no global spatial autocorrelation at the provincial level. The global spatial autocorrelation of CCC at the county level in Hubei province was significantly higher than that at the prefecture level. This paper presented only a case study of Hubei province, which could help to improve our understanding of spatiotemporal dynamic characteristics of the COVID-19 epidemic on the local scale. As the spread of the pandemic has been rapidly growing across the global, newer, bigger, and longer time series data might be collected and used to analyze the spatial statistics of the COVID-19 pandemic and to further expound its controlling mechanism from local to global scales.

Second, the Spearman’s rank correlation analysis of the influencing factors of the COVID-19 epidemic in Hubei province is only based on the relationship between different variable data, but not their cause and effect because neither individual infected data in detail nor contemporaneous data were collected for analysis. The spread of the COVID-19 epidemic is not only related to the natural environment, population mobility, and socioeconomic activities but also closely related to the policies of the epidemic prevention and control. This study explored the correlations between the number of confirmed COVID-19 cases and the natural, social, and economic indicators. Any study of the COVID-19 epidemic influencing factors requires further consideration of more detailed data of infectious cases, such as gender, age, job, etc., and more indicators of the natural environment, population, society, economy, medical conditions, and policies. Therefore, it could be beneficial to further study other potential influencing factors and in-depth epidemiology in order to reveal the causes of the COVID-19 epidemic spread.

Third, limited by the accessibility and availability of data, the results of the spatial statistics and influencing factors of the COVID-19 epidemic at the county scale in this study might have some uncertainties because of the lack of the county level data in Wuhan city and asymptomatic cases of COVID-19 not reported before [36]. Openness, transparency, and sharing of epidemic data are very important not only for epidemic prevention and control but also for scientific research [56,57]. Surprisingly, the data of COVID-19 cases were not publicly found at the county level in Wuhan city, where the COVID-19 epidemic was extensively severe during the early stage. It is desired to continue to update relevant data and methods to analyze spatiotemporal multi-scale evolution mechanisms of the COVID-19 epidemic and influencing factors of natural, social, economic, and medical indicators at the country, provincial, prefecture, and county levels (even townships and streets) in a more comprehensive and in-depth manner in order to provide more accurate and reliable support information for the scientific prevention and control of the COVID-19 pandemic.

## 5. Conclusions

In this paper, we established a new geodatabase for the COVID-19 epidemic study based on the COVID-19 epidemic data available publicly on multiple scales in Hubei province. Furthermore, we used ArcGIS spatial statistics methods to analyze spatiotemporal patterns of the COVID-19 epidemic and explored the influencing factors using Spearman’s rank correlation analysis from the prefectural and county levels in Hubei province, China during the early stage of the epidemic outbreak and spread from January to February 2020. The following conclusions were achieved.

(1) At both prefecture and county levels, extremely significant global spatial autocorrelations were observed for the number of CCC in Hubei from 30 January to 18 February 2020. The global spatial autocorrelation of the number of CCC at the county level was much stronger than that at the prefecture level in Hubei province, indicating a scale effect of this autocorrelation.

(2) Wuhan city and most of the districts/sub-cities in Wuhan city were significant high-risk epidemic areas at the prefecture, and county levels, respectively, during the study period.

(3) The numbers of CCC at both prefecture and county levels had local spatial autocorrelations at the 99% confidence and had positive and extremely significant correlations with main social and economic indicators (i.e., RGP, RSP, GDP, and TRS) from 30 January to 18 February 2020.

(4) The number of CCC had a moderate–strong, negative, and extremely significant correlation with major terrain parameters (i.e., MAXE, MNE, and RAE) at the county level, but no significant correlation at the prefecture level in Hubei province from 26 January to 18 February 2020.

In summary, the COVID-19 epidemics at both levels in Hubei province had the evident characteristics of significant global spatial autocorrelations, significant centralized outbreaks, and had an extremely significant association with social and economic factors during the certain study period. The natural factors, such as LA, MAXE, MNE, and RAE, had significant relationship with the COVID-19 epidemics at the county level, not at the prefecture level, from 2 February to 18 February 2020. In the meantime, the socioeconomic factors, such as RGP, RSP, TRS, and GDP, were significantly related to the COVID-19 epidemics at both levels from 26 January to 18 February 2020. Like repetitive experiments, the consistent time-series results of spatial autocorrelations and influencing factors should validate the reliability of our conclusions. It is expected that this study could help to improve our understanding of spatiotemporal patterns and influencing factors of the COVID-19 epidemic and benefit the scientific prevention and control of the epidemic for policymakers at multiple levels of government.

## Figures and Tables

**Table 1:** The Spearman’s rank correlation results of the number of cumulative confirmed COVID-19 cases (CCC) with the terrain (MINE, MAXE, MNE, and RAE), land area (LA), social (PD, RGP, RSP, and BMI), and economic (TRS and GDP) indicators at the prefecture level in Hubei province from 23 January to 18 February 2020.

Indicator	MINE	MAXE		MNE		RAE		LA		PD		RGP		RSP		TRS		GDP		BMI
CCC0123	−0.508 *	−0.084		−0.185		−0.097		0.218		0.231		0.640 **		0.647 **		0.555 *		0.608 **		0.579 *
CCC0124	−0.321	0.021		−0.067		0.018		0.328		0.123		0.650 **		0.605 *		0.411		0.418		0.460
CCC0125	−0.568 *	0.082		0.039		0.076		0.375		0.158		0.712 **		0.702 **		0.622 **		0.654 **		0.586 *
CCC0126	−0.515 *	0.113		0.075		0.104		0.417		0.169		0.757 **		0.765 **		0.689 **		0.737 **		0.602 *
CCC0127	−0.531 *	0.045		−0.006		0.037		0.347		0.254		0.753 **		0.764 **		0.699 **		0.766 **		0.704 **
CCC0128	−0.451	0.088		0.049		0.074		0.373		0.238		0.755 **		0.782 **		0.725 **		0.784 **		0.607 *
CCC0129	−0.468	0.047		−0.005		0.034		0.355		0.267		0.772 **		0.797 **		0.750 **		0.819 **		0.679 **
CCC0130	−0.473	−0.025		−0.061		−0.044		0.248		0.368		0.711 **		0.745 **		0.748 **		0.811 **		0.654 **
CCC0131	−0.468	0.022		−0.017		−0.002		0.316		0.319		0.765 **		0.799 **		0.811 **		0.865 **		0.668 **
CCC0201	−0.456	0.042		0.002		0.015		0.324		0.304		0.755 **		0.794 **		0.814 **		0.868 **		0.650 **
CCC0202	−0.527 *	−0.012		−0.054		−0.032		0.304		0.350		0.779 **		0.816 **		0.831 **		0.882 **		0.682 **
CCC0203	−0.505 *	0.034		0.005		0.010		0.326		0.304		0.767 **		0.794 **		0.806 **		0.850 **		0.661 **
CCC0204	−0.551 *	0.015		−0.022		0.000		0.324		0.348		0.799 **		0.824 **		0.838 **		0.875 **		0.725 **
CCC0205	−0.522 *	−0.027		−0.051		−0.051		0.265		0.370		0.738 **		0.760 **		0.782 **		0.824 **		0.675 **
CCC0206	−0.534 *	−0.059		−0.083		−0.086		0.233		0.395		0.721 **		0.748 **		0.770 **		0.816 **		0.657 **
CCC0207	−0.534 *	−0.059		−0.083		−0.086		0.233		0.395		0.721 **		0.748 **		0.770 **		0.816 **		0.657 **
CCC0208	−0.561 *	−0.074		−0.108		−0.096		0.228		0.439		0.750 **		0.775 **		0.804 **		0.843 **		0.732 **
CCC0209	−0.529 *	−0.071		−0.096		−0.100		0.208		0.419		0.708 **		0.733 **		0.760 **		0.804 **		0.675 **
CCC0210	−0.527 *	−0.096		−0.120		−0.125		0.174		0.449		0.689 **		0.711 **		0.735 **		0.782 **		0.686 **
CCC0211	−0.498 *	−0.086		−0.110		−0.115		0.189		0.436		0.691 **		0.718 **		0.745 **		0.787 **		0.657 **
CCC0212	−0.529 *	−0.120		−0.154		−0.145		0.172		0.451		0.696 **		0.718 **		0.725 **		0.787 **		0.704 **
CCC0213	−0.554 *	−0.147		−0.179		−0.172		0.127		0.490 *		0.667 **		0.684 **		0.701 **		0.757 **		0.725 **
CCC0214	−0.551 *	−0.135		−0.167		−0.162		0.137		0.485 *		0.674 **		0.691 **		0.716 **		0.767 **		0.732 **
CCC0215	−0.554 *	−0.147		−0.179		−0.172		0.127		0.490 *		0.667 **		0.684 **		0.701 **		0.757 **		0.725 **
CCC0216	−0.569 *	−0.162		−0.199		−0.184		0.108		0.517 *		0.659 **		0.676 **		0.699 **		0.755 **		0.754 **
CCC0217	−0.566 *	−0.150		−0.186		−0.174		0.118		0.512 *		0.667 **		0.684 **		0.713 **		0.765 **		0.761 **
CCC0218	−0.566 *	−0.150		−0.186		−0.174		0.118		0.512 *		0.667 **		0.684 **		0.713 **		0.765 **		0.761 **
tMean	−0.539 *	−0.113		−0.145		−0.140		0.169		0.466		0.701 **		0.721 **		0.743 **		0.792 **		0.725 **
N5	NES		NS		NM		NW		None		PW		PM		PS		PES		P5	
*p* < 0.05	−1~−0.8		−0.8~−0.6		−0.6~−0.4		−0.4~−0.2		−0.2~0.2		0.2~0.4		0.4~0.6		0.6~0.8		0.8~1		*p* < 0.05	

Notes for the correlation coefficient ranking of Spearman’s ρ (*p* < 0.01): NES, negative and extremely strong; NS, negative and strong; NM, negative and moderate; NW, negative weak; None, not significant; PW, positive and weak; PM, positive and moderate; PS, positive and strong; PES, positive and extremely strong; N5, negative correlation (*p* < 0.05); P5, positive correlation (*p* < 0.05). ** indicates that the correlation is significant when the confidence (double test) is 0.01; * indicates that the correlation is significant when the confidence (double test) is 0.05. CCC0123 denotes the number of cumulative confirmed cases on 23 January 2020, and the like. tMean denotes the average of the cumulative confirmed COVID-19 cases from 23 January to 18 February 2020. MINE denotes minimum elevation. MAXE denotes maximum elevation. MNE denotes the mean of elevation. RAE denotes a range of elevations. LA denotes land area. PD denotes population density. RGP denotes registered population. RSP denotes the resident population. TRS denotes the total retail sales of consumer goods. GDP denotes regional gross domestic product. Moreover, BMI denotes the Baidu migration index.

**Table 2:** The Spearman’s rank correlation results of the number of cumulative confirmed COVID-19 cases with the terrain (MINE, MAXE, MNE, and RAE), land area (LA), social (PD, RGP, and RSP), and economic (TRS and GDP) indicators at the county level in Hubei province from 26 January to 18 February 2020.

Indicator	MINE	MAXE	MNE	RAE	LA	PD	RGP	RSP	TRS	GDP
CCC0126	−0.314 *	−0.477 **	−0.513 **	−0.478 **	−0.289 *	0.482 **	0.257 *	0.286 *	0.449 **	0.290 *
CCC0127	−0.287 *	−0.537 **	−0.523 **	−0.529 **	−0.150	0.424 **	0.344 **	0.386 **	0.470 **	0.331 **
CCC0128	−0.321 **	−0.483 **	−0.484 **	−0.482 **	−0.179	0.499 **	0.466 **	0.508 **	0.591 **	0.488 **
CCC0129	−0.326 **	−0.491 **	−0.494 **	−0.489 **	−0.145	0.526 **	0.529 **	0.575 **	0.648 **	0.538 **
CCC0130	−0.354 **	−0.537 **	−0.534 **	−0.535 **	−0.221 *	0.583 **	0.499 **	0.583 **	0.705 **	0.633 **
CCC0131	−0.372 **	−0.557 **	−0.544 **	−0.556 **	−0.266 *	0.613 **	0.465 **	0.552 **	0.704 **	0.622 **
CCC0201	−0.406 **	−0.532 **	−0.552 **	−0.526 **	−0.254 *	0.618 **	0.494 **	0.578 **	0.705 **	0.609 **
CCC0202	−0.456 **	−0.570 **	−0.601 **	−0.561 **	−0.276 **	0.657 **	0.530 **	0.613 **	0.706 **	0.597 **
CCC0203	−0.488 **	−0.589 **	−0.628 **	−0.577 **	−0.277 **	0.664 **	0.547 **	0.630 **	0.712 **	0.606 **
CCC0204	−0.502 **	−0.603 **	−0.640 **	−0.590 **	−0.305 **	0.691 **	0.545 **	0.626 **	0.699 **	0.586 **
CCC0205	−0.509 **	−0.611 **	−0.649 **	−0.598 **	−0.311 **	0.695 **	0.543 **	0.624 **	0.696 **	0.589 **
CCC0206	−0.511 **	−0.614 **	−0.651 **	−0.600 **	−0.293 **	0.689 **	0.553 **	0.634 **	0.694 **	0.584 **
CCC0207	−0.517 **	−0.624 **	−0.665 **	−0.610 **	−0.297 **	0.696 **	0.553 **	0.635 **	0.703 **	0.584 **
CCC0208	−0.519 **	−0.631 **	−0.669 **	−0.617 **	−0.299 **	0.700 **	0.554 **	0.638 **	0.705 **	0.584 **
CCC0209	−0.520 **	−0.632 **	−0.668 **	−0.619 **	−0.299 **	0.703 **	0.554 **	0.636 **	0.696 **	0.571 **
CCC0210	−0.518 **	−0.633 **	−0.668 **	−0.619 **	−0.295 **	0.700 **	0.551 **	0.632 **	0.697 **	0.566 **
CCC0211	−0.522 **	−0.642 **	−0.679 **	−0.629 **	−0.292 **	0.706 **	0.561 **	0.642 **	0.705 **	0.570 **
CCC0212	−0.525 **	−0.646 **	−0.680 **	−0.634 **	−0.269 *	0.689 **	0.570 **	0.650 **	0.705 **	0.577 **
CCC0213	−0.528 **	−0.632 **	−0.677 **	−0.620 **	−0.284 **	0.694 **	0.575 **	0.648 **	0.694 **	0.560 **
CCC0214	−0.533 **	−0.635 **	−0.683 **	−0.622 **	−0.283 **	0.690 **	0.570 **	0.642 **	0.696 **	0.559 **
CCC0215	−0.534 **	−0.640 **	−0.687 **	−0.627 **	−0.277 **	0.689 **	0.580 **	0.653 **	0.704 **	0.567 **
CCC0216	−0.532 **	−0.646 **	−0.690 **	−0.632 **	−0.277 **	0.690 **	0.579 **	0.651 **	0.704 **	0.569 **
CCC0217	−0.530 **	−0.650 **	−0.693 **	−0.636 **	−0.276 **	0.690 **	0.580 **	0.652 **	0.710 **	0.574 **
CCC0218	−0.525 **	−0.650 **	−0.690 **	−0.638 **	−0.275 **	0.688 **	0.574 **	0.649 **	0.708 **	0.572 **
tMean	−0.515 **	−0.638 **	−0.677 **	−0.626 **	−0.290 **	0.702 **	0.562 **	0.645 **	0.720 **	0.587 **
N5	NES	NS	NM	NW	None	PW	PM	PS	PES	P5
*p* < 0.05	−1~−0.8	−0.8~−0.6	−0.6~−0.4	−0.4~−0.2	−0.2~0.2	0.2~0.4	0.4~0.6	0.6~0.8	0.8~1	*p* < 0.05

Notes for the correlation coefficient ranking of Spearman’s ρ (*p* < 0.01): NES, negative and extremely strong; NS, negative and strong; NM, negative and moderate; NW, negative weak; None, not significant; PW, positive and weak; PM, positive and moderate; PS, positive and strong; PES, positive and extremely strong; N5, negative correlation (*p* < 0.05); P5, positive correlation (*p* < 0.05). ** indicates that the correlation is significant when the confidence (double test) is 0.01; * indicates that the correlation is significant when the confidence (double test) is 0.05. CCC0126 denotes the number of cumulative confirmed cases on 26 January 2020, and the like. tMean denotes the average of the cumulative confirmed COVID-19 cases from 26 January to 18 February 2020. MINE denotes minimum elevation. MAXE denotes maximum elevation. MNE denotes the mean of elevation. RAE denotes a range of elevations. LA denotes land area. PD denotes population density. RGP denotes registered population. RSP denotes the resident population. TRS denotes the total retail sales of consumer goods. Moreover, GDP denotes regional gross domestic product.

## Supplementary Materials

The following are available online at https://www.mdpi.com/1660-4601/17/11/3903/s1, Table S1: Summary of the Spearman’s rank correlation results for the daily new confirmed COVID-19 cases with the terrain, land area, social, and economic indicators, and Baidu migration index at the prefecture level in Hubei from 24 January to 18 February 2020, Table S2: Summary of the Spearman’s rank correlation results for the number of the daily new confirmed COVID-19 cases with the land area, terrain, social, and economic indicators, respectively, at the county level in Hubei province from 26 January to 18 February 2020. The datasets used and analyzed during the current study are available from the corresponding author on reasonable request.

## Abbreviations

SARS	severe acute respiratory syndrome
SARS-CoV-2	2019 novel coronavirus
COVID-19	novel coronavirus pneumonia 2019 (coronavirus disease 2019)
CCC	cumulative confirmed COVID-19 cases
DCC	daily new confirmed COVID-19 cases
LISA	Local Indicators of Spatial Association
ALMI	Anselin Local Moran’s I
LA	land area
PD	population density
RGP	registered population
RSP	resident population
BMI	Baidu migration index
GDP	gross domestic production
TRS	total retail sales of consumer goods
DEM	digital elevation model
MAXE	maximum elevation
MINE	minimum elevation
MNE	mean elevation
RAE	range of elevation
