# Supplementary material for: Spatial Statistics and Influencing Factors of the COVID-19 Epidemic at Both Prefecture and County Levels in Hubei Province, China
Source: Int J Environ Res Public Health. 2020 May 31;17(11):3903. doi: 10.3390/ijerph17113903 (PMC7312640; doi:10.3390/ijerph17113903)
Supplement: Supplementary file 1 [file ijerph-17-03903-s001.pdf]

Table S1. Summary of the Spearman's rank correlation results for the new confirmed COVID-19 cases with the terrain, land area, population and economic indicators, and Baidu migration index at the prefecture level in Hubei.

Indicator	MINE	MAXE	MNE	RAE	LA	PD	RGP	RSP	TRS	GDP	BDMI
ncc0124	-0.173	0.021	-0.044	0.031	0.263	0.040	0.480	0.440	0.235	0.242	0.190
ncc0125	-0.565*	0.119	0.106	0.119	0.407	0.148	0.713**	0.723**	0.706**	0.708**	0.485
ncc0126	-0.330	0.087	0.038	0.070	0.355	0.206	0.672**	0.712**	0.647**	0.720**	0.487
ncc0127	-0.424	0.016	-0.055	-0.011	0.242	0.380	0.688**	0.717**	0.708**	0.761**	0.767**
ncc0128	-0.434	0.059	0.032	0.044	0.289	0.348	0.733**	0.750**	0.733**	0.752**	0.643**
ncc0129	-0.526*	0.013	-0.058	0.007	0.329	0.312	0.748**	0.795**	0.831**	0.874**	0.692**
ncc0130	-0.439	-0.091	-0.112	-0.128	0.142	0.454	0.653**	0.680**	0.723**	0.774**	0.647**
ncc0131	-0.553*	0.034	0.016	0.007	0.311	0.305	0.727**	0.757**	0.796**	0.835**	0.624*
ncc0201	-0.411	-0.028	-0.075	-0.056	0.232	0.342	0.671**	0.717**	0.733**	0.801**	0.581*
ncc0202	-0.528*	-0.022	-0.067	-0.044	0.272	0.380	0.780**	0.791**	0.784**	0.791**	0.722**
ncc0203	-0.466	0.042	0.029	0.012	0.311	0.272	0.728**	0.750**	0.777**	0.824**	0.607*
ncc0204	-0.539*	-0.120	-0.159	-0.142	0.130	0.522*	0.681**	0.696**	0.745**	0.757**	0.754**
ncc0205	-0.541*	-0.155	-0.183	-0.179	0.159	0.396	0.634**	0.673**	0.700**	0.771**	0.545*
ncc0206	-0.591*	-0.136	-0.164	-0.158	0.146	0.487*	0.687**	0.703**	0.732**	0.775**	0.727**
ncc0207	-0.468	-0.272	-0.333	-0.294	-0.039	0.637**	0.556*	0.588*	0.620**	0.654**	0.686**
ncc0208	-0.498*	-0.076	-0.132	-0.096	0.184	0.485*	0.681**	0.738**	0.809**	0.838**	0.671**
ncc0209	-0.468	-0.253	-0.294	-0.282	-0.027	0.587*	0.538*	0.531*	0.544*	0.542*	0.622*
ncc0210	-0.323	-0.250	-0.286	-0.296	-0.041	0.603*	0.508*	0.537*	0.523*	0.530*	0.442
ncc0211	-0.527*	-0.319	-0.359	-0.330	-0.097	0.627**	0.443	0.472	0.477	0.522*	0.514*
ncc0212	-0.616**	-0.429	-0.454	-0.401	-0.125	0.607**	0.401	0.385	0.376	0.412	0.416
ncc0213	-0.392	-0.080	-0.126	-0.115	0.048	0.320	0.380	0.389	0.370	0.374	0.368
ncc0214	-0.632**	-0.274	-0.276	-0.288	-0.108	0.588*	0.400	0.375	0.472	0.514*	0.698**
ncc0215	-0.218	-0.612**	-0.578*	-0.653**	-0.442	0.692**	0.097	0.091	0.112	0.213	0.109
ncc0216	0.015	-0.561*	-0.552*	-0.623**	-0.383	0.631**	0.117	0.114	0.031	0.077	-0.093
ncc0217	0.010	-0.469	-0.463	-0.514*	-0.275	0.568*	0.150	0.156	0.212	0.193	-0.002
ncc0218	-0.050	-0.139	-0.221	-0.189	0.048	0.292	0.446	0.490*	0.489*	0.514*	0.329
nMean	-0.566*	-0.150	-0.186	-0.174	0.118	0.512*	0.667**	0.684**	0.713**	0.765**	0.761**

N5	NES	NS	NM	NW	None	PW	PM	PS	PES	P5
p<0.05	-1 ~ -0.8	-0.8 ~ -0.6	-0.6 ~ -0.4	-0.4 ~ -0.2	-0.2 ~ 0.2	0.2 ~ 0.4	0.4 ~ 0.6	0.6 ~ 0.8	0.8 ~ 1	*p*<0.05

Legend for the correlation coefficient ranking of Spearman's ρ (p<0.01): NES, negative and extremely strong; NS, negative and strong; NM, negative and moderate; NW, negative weak; None, not significant; PW, positive and weak; PM, positive and moderate; PS, positive and strong; PES, positive and extremely strong; N5, negative correlation (p<0.05); P5, positive correlation (p<0.05). ** indicates that the correlation is significant when the confidence (double test) is 0.01; * indicates that the correlation is significant when the confidence (double test) is 0.05. ncc0124 indicates the number of new confirmed cases and so on. nMean indicates to the average of the cumulative confirmed COVID-19 cases from January 24 to February 18, 2020. MINE indicates minimum elevation. MAXE indicates maximum elevation. MNE indicates mean of elevation. RAE indicates range of elevation. LA indicates land area. PD indicates population density. RGP indicates registered population. RSP indicates resident population. TRS indicates total retail sales of consumer goods. GDP indicates regional gross domestic product. And BDMI indicates Baidu migration index.

Table S2. Summary of the Spearman's rank correlation results for the number of new confirmed COVID-19 cases with the land area, terrain, social, and economic indicators, respectively, at the county level in Hubei province from January 26 to February 18, 2020.

**Indicator**	**MINE**	**MAXE**	**MNE**	**RAE**	**LA**	**PD**	**RGP**	**RSP**	**TRS**	**GDP**
ncc0126	-0.11	-0.276**	-0.212*	-0.297**	-0.278**	0.335**	0.049	0.076	0.217*	0.111
ncc0127	-0.355**	-0.488**	-0.419**	-0.483**	-0.237*	0.379**	0.193	0.236*	0.366**	0.409**
ncc0128	-0.254*	-0.350**	-0.327**	-0.348**	-0.017	0.315**	0.534**	0.538**	0.440**	0.303**
ncc0129	-0.327**	-0.460**	-0.480**	-0.459**	-0.199	0.423**	0.291**	0.333**	0.448**	0.335**
ncc0130	-0.171	-0.385**	-0.382**	-0.383**	-0.119	0.451**	0.355**	0.458**	0.611**	0.548**
ncc0131	-0.235*	-0.407**	-0.346**	-0.415**	-0.346**	0.511**	0.155	0.233*	0.442**	0.361**
ncc0201	-0.296**	-0.389**	-0.403**	-0.380**	-0.169	0.457**	0.329**	0.401**	0.506**	0.427**
ncc0202	-0.480**	-0.511**	-0.574**	-0.490**	-0.297**	0.626**	0.484**	0.533**	0.547**	0.420**
ncc0203	-0.426**	-0.483**	-0.515**	-0.466**	-0.183	0.507**	0.493**	0.555**	0.545**	0.503**
ncc0204	-0.434**	-0.531**	-0.552**	-0.524**	-0.332**	0.663**	0.479**	0.534**	0.547**	0.406**
ncc0205	-0.403**	-0.532**	-0.531**	-0.518**	-0.212*	0.532**	0.458**	0.528**	0.565**	0.530**
ncc0206	-0.397**	-0.501**	-0.533**	-0.488**	-0.131	0.505**	0.464**	0.545**	0.545**	0.407**
ncc0207	-0.470**	-0.571**	-0.636**	-0.562**	-0.328**	0.648**	0.419**	0.497**	0.611**	0.457**
ncc0208	-0.456**	-0.601**	-0.596**	-0.598**	-0.315**	0.633**	0.319**	0.422**	0.593**	0.460**
ncc0209	-0.399**	-0.399**	-0.463**	-0.383**	-0.105	0.431**	0.446**	0.448**	0.356**	0.207
ncc0210	-0.296**	-0.492**	-0.488**	-0.491**	-0.208	0.555**	0.382**	0.447**	0.548**	0.369**
ncc0211	-0.362**	-0.460**	-0.494**	-0.458**	-0.191	0.501**	0.326**	0.372**	0.418**	0.271*
ncc0212	-0.385**	-0.576**	-0.566**	-0.580**	-0.193	0.527**	0.351**	0.408**	0.504**	0.344**
ncc0213	-0.231*	-0.172	-0.309**	-0.155	-0.223*	0.349**	0.250*	0.255*	0.234*	0.153
ncc0214	-0.321**	-0.406**	-0.396**	-0.392**	-0.255*	0.400**	0.13	0.176	0.358**	0.284**
ncc0215	-0.179	-0.391**	-0.430**	-0.395**	-0.133	0.334**	0.252*	0.263*	0.309**	0.236*
ncc0216	-0.133	-0.411**	-0.382**	-0.421**	-0.222*	0.371**	0.143	0.184	0.353**	0.285**
ncc0217	-0.132	-0.514**	-0.457**	-0.526**	-0.265*	0.472**	0.190	0.268*	0.423**	0.348**
ncc0218	-0.175	-0.293**	-0.266*	-0.294**	-0.17	0.307**	0.073	0.145	0.211*	0.082
nMean	-0.521**	-0.651**	-0.689**	-0.637**	-0.276**	0.687**	0.569**	0.644**	0.702**	0.567**

N5	NES	NS	NM	NW	None	PW	PM	PS	PES	P5
p<0.05	-1 ~ -0.8	-0.8 ~ -0.6	-0.6 ~ -0.4	-0.4 ~ -0.2	-0.2 ~ 0.2	0.2 ~ 0.4	0.4 ~ 0.6	0.6 ~ 0.8	0.8 ~ 1	*p*<0.05

Legend for the correlation coefficient ranking of Spearman's ρ ($p<0.01$): NES, negative and extremely strong; NS, negative and strong; NM, negative and moderate; NW, negative weak; None, not significant; PW, positive and weak; PM, positive and moderate; PS, positive and strong; PES, positive and extremely strong; N5, negative correlation ($p<0.05$); P5, positive correlation ($p<0.05$). ** indicates that the correlation is significant when the confidence (double test) is 0.01; * indicates that the correlation is significant when the confidence (double test) is 0.05. ncc0126 indicates the number of new confirmed cases and so on. nMean indicates to the average of the cumulative confirmed COVID-19 cases from January 26 to February 18, 2020. MINE indicates minimum elevation. MAXE indicates maximum elevation. MNE indicates mean of elevation. RAE indicates range of elevation. LA indicates land area. PD indicates population density. RGP indicates registered population. RSP indicates resident population. TRS indicates total retail sales of consumer goods. And GDP indicates gross domestic product.
